# Supplementary material for: Narrative Voice Matters! Improving Smoking Prevention with Testimonial Messages through Identification and Cognitive Processes
Source: Int J Environ Res Public Health. 2020 Oct 5;17(19):7281. doi: 10.3390/ijerph17197281 (PMC7579370; doi:10.3390/ijerph17197281)
Supplement: Supplementary file 1 [file ijerph-17-07281-s001.pdf]

Supplementary File S1

Narratives

(a) First-person narrative [message type]

My name is Teresa, and I am 45 years old. I have been a smoker for 20 years but have not smoked for over a year. I have tried to quit smoking before, three times [I have not tried to quit before, this was my first time].

A little over a year ago, I began to notice a strong pain when chewing and even felt as if my teeth were moving. Also, my gums were very swollen, and when I brushed my teeth they always bled. I decided to go to the dentist, and he said that I had periodontitis, better known as pyorrhoea. He explained that it was not very advanced and that, if I stopped smoking, I could fully recover. The truth is that I didn't think it was that serious until he told me that, if I didn't stop smoking, I could lose all my teeth.

I will not lie, those were very hard days, with very strange feelings. I remember that during the first few weeks I had a lot of anxiety, to the point that sometimes I felt like I was drowning. The worry, nerves and lack of concentration were terrible, but the worst was the sadness, the exhaustion and the constant crying. I often thought about smoking a cigarette. "One and that's it," I said to myself, but then I remembered that I was sick because of tobacco, and my desire to smoke went away. After all, this was my fourth attempt to quit smoking [After all, this was my first attempt to quit smoking]. Everything improved after the third or fourth month. My symptoms had almost disappeared thanks to the antibiotics, but my dentist recommended that I undergo treatment to remove the tartar deeper under my gums, to get rid of the disease completely. When I quit smoking, my body responded perfectly: the inflammation, bleeding and discomfort when chewing disappeared, my breath no longer smelled bad and my teeth were much whiter. Now even food tastes much better to me! Also, at the beginning, I bought a piggy bank, and every day I put in what I was going to spend on tobacco and used it to go out or to buy myself treats; I was spurred on by seeing how my health improved while I also saved a lot of money.

I was able to quit tobacco after several attempts [I was able to quit tobacco on my first attempt]. Although it was hard, now I can only say that it was worth it. I can no longer imagine puffing on a cigar, not after seeing how good I feel after quitting. The urge to smoke has disappeared, and my gums and health have improved. All this makes me not want to smoke again: I won't let tobacco harm my health again.

(b) Third-person narrative [message type]

Teresa is 45 years old. She has been a smoker for 20 years but has not smoked for more than a year. She had already tried to quit smoking before, three times [She had not tried to quit before; this was her first time].

A little over a year ago, she began to notice a strong pain when chewing and even felt as if her teeth were moving. Also, her gums were very swollen, and when she brushed her teeth they always bled. Teresa decided to go to the dentist, who told her that she had periodontitis, better known as pyorrhoea. He explained that it was not very advanced and that, if she stopped smoking, she could fully recover. The truth is that Teresa did not think it was so serious until she told him that, if she did not stop smoking, she could lose all her teeth.

Those were very hard days for Teresa, with very strange feelings. For the first few weeks, she was very anxious, to the point that sometimes she felt like she was drowning. The worry, nerves and lack of concentration were terrible, but the worst was the sadness, the exhaustion and the constant crying. On many occasions she thought about smoking a cigarette. "One and that's it," she told herself, but then she remembered that she was ill because of tobacco, and her desire to smoke went away. After all, this was her fourth attempt to quit smoking [After all, this was her first attempt to quit smoking]. Everything improved from the third or fourth month. The symptoms had almost

disappeared thanks to the antibiotics, but her dentist recommended that she undergo treatment to remove the tartar deeper under the gums, to get rid of the disease completely. When she quit smoking, her body responded perfectly: the inflammation, bleeding and discomfort when chewing disappeared, her breath no longer smelled bad and her teeth were much whiter. Now even food tastes much better to her! In addition, at the beginning, she bought a piggy bank and every day put in what she was going to spend on tobacco and used it to go out or buy herself treats; This spurred her on as she saw how her health improved while at the same time saving a lot of money.

She was able to quit tobacco after several attempts [She was able to quit tobacco on her first attempt]. Although it was hard, now she can only say that it was worth it. She no longer imagines puffing on a cigar, not after seeing how good she feels after quitting. Teresa’s desire to smoke has disappeared, and her gums and health have improved. All this makes her not want to smoke again: she won’t let tobacco harm her health again.
